# Prospective double-blind clinical trial evaluating the effectiveness of Bromelain in the third molar extraction postoperative period

**DOI:** 10.4317/medoral.19105

**Published:** 2013-12-07

**Authors:** María C. de la Barrera-Núñez, Rosa M. Yáñez-Vico, Antonio Batista-Cruzado, Jean M. Heurtebise-Saavedra, Raquel Castillo-de Oyagüe, Daniel Torres-Lagares

**Affiliations:** 1 DDS, Departament of Stomatology. School of Dentistry. University of Seville; 2 DDS, PhD, Departament of Stomatology. School of Dentistry. University of Seville; 3 DDS, PhD, Departament of Stomatology. School of Dentistry. University Complutense; 4 DDS, PhD, Professor of Oral Surgery. Department of Stomatology. University of Seville

## Abstract

Objectives: To evaluate the anti-inflammatory and analgesic effect of Bromelain (pineapple extract) administered orally in the postoperative after extraction of impacted lower molars. Study Design: This is a prospective, placebo-controlled, unicentric, double-blind study; the sample size was 34 patients. The pre and postoperative outcomes, evaluated on the third (D3) and eighth day (D8), included inflamtion, pain and oral aperture, as well as the need for analgesics. One group received bromelain 150mg per day for three days and 100mg on days 4 to 7. The other group received placebo in the same dosage. All outcomes werrecorded quantitatively and analyzed with the Mann-Whitney U test for independent samples. Results: Although there were no statistically significant differences between the treatment groups, a trend towards less inflammation and improved oral aperture was observed in the group that received bromelain, compared to the group that received placebo. This trend can be attributed completely to random reasons, since there is no statistical difference in the results. Conclusions: Further studies are necessary to analyze different administration patterns and doses of bromelain for the use in the postoperative of impacted third molars.

** Key words:**Tooth extraction, third molar, postoperative period, bromeline, clinical study.

## Introduction

Bromelain (pineapple enzyme, Ananas comasus) is an aqueous extract obtained from the stem and fruit of the pineapple plant that contains high levels of proteolytic enzymes and which composition varies depending on the source and purification method (1,2). Bromelain directly influences pain mediators such as bradykinin (3), although its analgesic properties are closely linked to its anti-inflammatory properties (4,5). It has beenshownthat this fibrinolytic agent promotes reabsorption of edema in the blood circulation (6); it reduces swelling, bruising, pain, and healing time after trauma and surgical procedures (5). Evidence has shown that bromelain digests fibrin, thus allowing the elimination of edema (7). Indirectly, bromelain increases the time required for the conversion of prothrombin into thrombin, and thus activating plasminogen into plasmin; by these means, it prevents the formation of fibrin (7). All these cause a reduction in vascular permeability. In addition, bromelain inhibits the synthesis of pro-inflammatory prostaglandins, particularly PGE2 (8).

The most common postoperative complications after the extraction of an impacted wisdom tooth are a direct consequence of the inflammatory response to the surgical procedure (9) and include pain, inflammation and difficulty opening the mouth (10). Generally this response is controlled with the surgical technique (11-16) and mainly with pharmacological anti-inflammatory measures (17,18).

The purpose of our study was to assess the effect of bromelain on inflammation, pain perception, and trismus,after the extraction of an impacted lower third molar, using a prospective double-blind clinical trial.

## Material and Methods

-Subjects

34 healthy subjects,patients from the Faculty of Odontology of the University of Seville (Spain) who needed the extraction of a lower third molar, were consecutively selected for the study. All the extractions were classified with a Koerner index (19) 5-6 (moderately difficult). This way, we excluded all candidates who met any exclusion criteria: allergy to any of the components used in the study;contraindication for the administration of adrenaline; psychiatric treatment; pregnancy or lactation; coagulation disorders or any treatment that may alter it; antibiotic treatment, analgesics or anti-inflammatories used on the four days prior to the intervention; active periodontitis; smoking more than ten cigarettes per day. The eligible patients were informed of the study and gave their consent. The University of Seville Research Ethics Committeeindependently approved the procedure.

-Surgical Procedure

Articaine 4% with adrenaline 1:100.000was used as anesthetic. In all cases a bayonet incision with vertical discharge was performed on the mesial half of the second molar;a mucoperiosteal flap was lifted, and osteotomy was performed with a number eight tungsten carbide bur mounted on a hand piece; the extraction was completed by drift. Odontosection was performed with turbine and fissure bur, when needed.

The material used to suture the surgical wound was black braided silk 4/0, needle holder, toothless Adson forceps and scissors. The same operator performed all the interventions, and the same surgical material was used. The surgical protocol was not modified in any case.

-Pharmacological Treatment

It was decided to include a total of 40 patients in the study (20 per group), considering that the difference in inflammation marked on the third postoperative day would be 1.25 cm between the two groups, and estimating a variance of 2, with an alpha error of 0.05 (bila-teral) and a beta error of 0.2.

**Figure 1:**
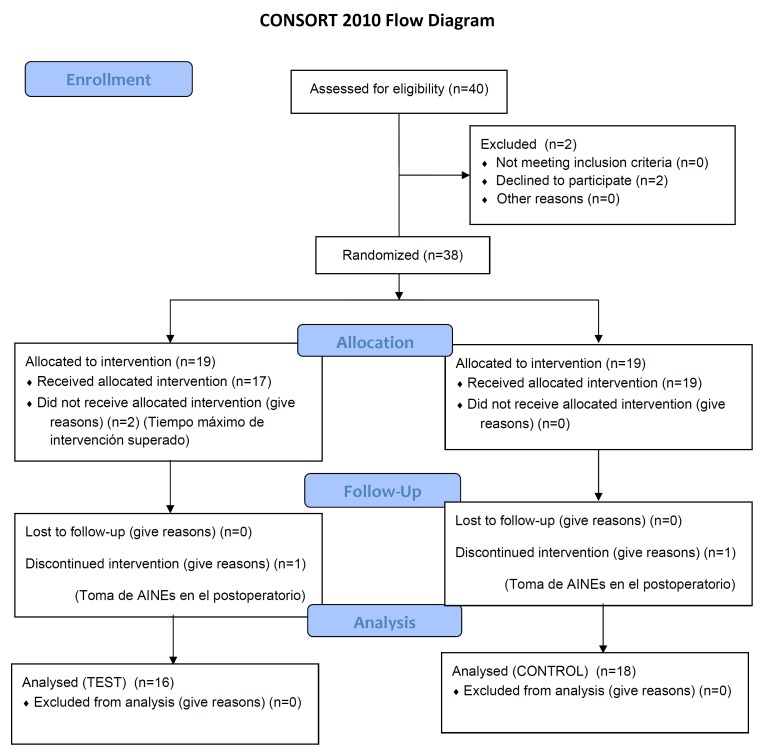
Consort flow chart.

For various reasons, the analysis was limited to 34 subjects (Fig. 1). After maintaining the same alpha and beta errors, this sample size should be enough to identify a 1.4 cm difference in swelling between the two groups, marked on the third postoperative day, and estimating a variance of 2.

The analysis was performed following a random and independent division on the side (right or left) into two groups: Test group (n = 16) and Control group (n = 18) (Fig. 1). All patients received the following medications: a) amoxicillin / clavulanate acid 875/125mg every 8 hours for 7 days, b) Paracetamol 500 mg (as first step rescue); Nolotil ® 575 mg (Boehringer Ingelheim, Spain, SA Sant Cugat del Valles, Barcelona) (as second step rescue). To assess the effect of bromelain, the tablets used in this trialcontained 100 nkat, equivalent to 50mg of Ananas stem. The Test group was administered a regimen of three tablets per day during the first three days and two tablets per day from the fourth to the seventh day. The Control Group was given placebo, three tablets a day for the first three days and two tablets a day from the fourth to the seventh day. When corticosteroid administration was required, following tooth extraction with a large osteotomy and / or long duration (over 30 minutes), the patient was excluded from the study due to the increased inflammation that both produce. In our study, two patients were excludedfor this reason; and two patients were excluded due to the administration of anti-inflammatory drugs that were not included in the study.

The randomization was performed through a randomized list generated on a computer by the company Rothapharm, which used this randomization to prepare the pharmacological blisters of placebo or medication, with a blind design that did not allow the identification of any of them without access to the list; this access was only at the end of the study to analyze the data.

-Assessment of postoperative pain

Postoperative pain was calculated using a Visual Analogue Scale (VAS) of 10 cm in length, that is, a straight line with endpoints representing the sensation limits: no pain in one end and unimaginable pain at the other (20). The patient registered the pain values presented at the beginning of the study, on day 3 (D3) and on day 8 (D8) postoperatively. We used a range of 0-10 (cm), with 0 beingabsolute absence of pain and 10 the maximum degree of pain.

-Assessment of postoperative facial swelling

Postoperative swelling was calculated using a Visual Analogue Scale (VAS) of 10 cm in length, that is, a straight line which endpoints represent the sensation limits: no swelling at one end and maximum swelling at the other (20). The patient registered the swelling values experienced at the beginning of the study (D0), on day 3 (D3) and on day 8 (D8) postoperatively. We used a range of 0-10 (cm), with 0 being absolute absence of swelling and 10 the maximum degree of swelling.

-Assessment of postoperative trismus

**Figure 2:**
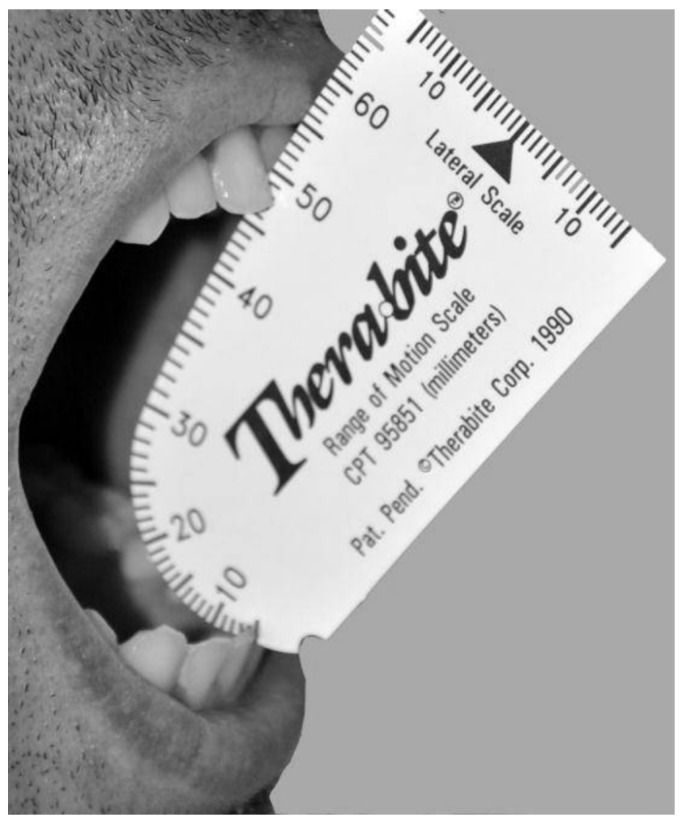
Quantification method of oral aperture from mesial-incisal angle of lower central incisor to mesial-incisal angle of maxillary central incisor.

The degree of trismus was assessed by measuring the interincisal distance before surgery (D0), on the 3rd (D3), and 8th (D8) days postoperatively, with a specific ruler for oral aperture measurement (Therabite ®). The results are expressed in mm (Fig. 2).

-Statistical analysis

The data was analyzed using SPSS 17.0 software for Windows (LEAD Technologies, USA). Data analysis consisted of descriptive analysis (mean and standard deviation) of the quantitative variables (age, inflammation, pain and aperture). The differences between the two groups for each one of the variables was performed using the Mann-Whitney U test for independent samples with significant differences if p <0.05.

## Results

The total number of patients studied was 17 men and 17 women with a mean age of 23.82 ± 3.9 years. In the control group 10 men and 7 women were treated, with an average age of 24.67 ± 4.2 years. In the experimental group 6 men and 11 womenwere treated, with a median age of 22.88 ± 3.6 years. Differences in age in both groups were not significant (p ≥ 0.05).

Preoperatively, the measurement of swelling, pain and aperture in both groups was similar, except for the inflammation which was slightly higher in the experimental group, although no statistically significant differences existed (p <0.05) in any of them ( Table 1).

**Table 1:**
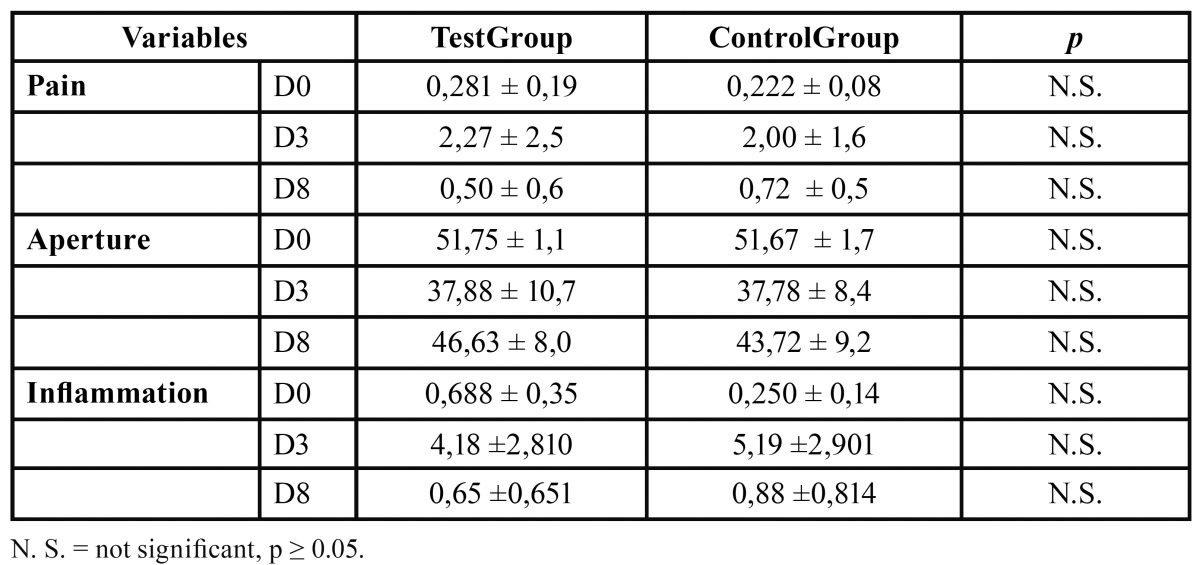
Preoperative (D0), and postoperative measurements (D3 = Day 3; D8 = day 8) in mm (mean ± d.e) in both study groups, comparisons by Mann-Whitney U test.

 Table 1 shows the results of the comparison between the control group and the test group on days 3 and 8, in the different postoperative outcomes. No significant diffe-rences were observed in the assessment of pain between the two treatment groups.

Regarding measurement of the oral aperture, one can see that after D3 and specifically in the evaluation at the end of treatment on D8, there is a tendency to an increased aperture (3 mm more then the average) in the group receiving bromelain, although the results were not significant.

The biggest effect of bromelain was observed in inflammation. Patients in the bromelain group, started from a slightly higher swelling values and at the end treatment, there was a slight tendency to a reduction in inflammation (0.65 ± 0.6 vs. 0.88 ± 0.8), not reaching statistical or clinical significance.

**Figure 3:**
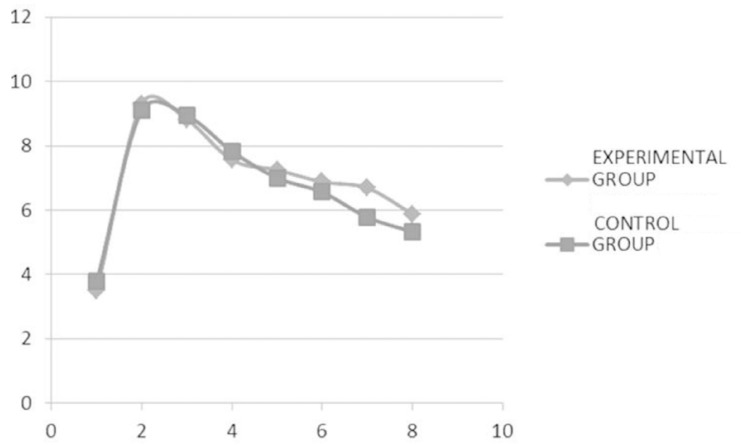
Quantification method of oral aperture from mesial-incisal angle of lower central incisor to mesial-incisal angle of maxillary central incisor.

Regarding the postoperative use of analgesic medications by patients, there was an increase in the administration between days 1 and 3, after which there was a progressive decrease (Fig. 3). When comparing both study groups, we found no significant differences in the use of medication during the first eight postoperative days (although it was slightly lower in the control group).

## Discussion

Several studies support the usefulness of orally administered bromelain to reduce pain and swelling after certain surgical procedures (4,5) due to a decreased level of certain pain mediators and vascular phenomena associated with acute inflammation (3,6).

Proteases, such as bromelain, act at specific points on the cell surface of the inflammatory component, specifically where their receptors are found, generating and / or destroying receptor agonists and activating and deactivating them, so they contribute significantly in the signal transduction mechanism of the inflammation. However, this effect can be highly variable depending on the different levels of expression of these receptors, not only in different individuals, but also in different tissues of the same individual (21).

Most of the published studies suggest various types of mechanisms by which proteases, including bromelain, can decrease inflammation. Among them the most prominent are:

-Favoring the return of the interstitial fluid and inflammatory component cells into the bloodstream, and thus decreasing the swelling of the area (22)

-Decreasing the biosynthesisof plasmacinins and proinflammatory prostaglandins PGE2, PGF2 (23)

In our study we have evaluated the efficacy of bromelain in inflammation, pain and degree of oral aperture after the extraction of a lower third molar. Our results are consistent with those obtained by Hozt et al. (24) who evaluated the anti-inflammatory effect of bromelain after the extraction of third molars administering bromelain 80mg 3 times a day for 6 days. In our study we used an oral administration of 150 mg, whereas Hozt et al. used 240 mg. According to the published results, the administration of 150mg a day does not seem to be the most effective regimen; or at least it is less effective than the one used by Hozt et al.

Although the results obtained were similar between the two groups regarding pain and inflammation, they (24) obtained a reduced need for analgesic intake in the group that was administered bromelain. This last fact has not been confirmed in our study, as the use of medication in both groups was very similar.

On the other hand, Inchigolo et al. (25) compared the use of bromelain (40 mg every 6 hours for 6 days) with ketoprofen (100mg every 12 hours for six days, and found no differences between them. Therefore, they concluded that bromelain is as effective as an NSAID in the treatment of postoperative inflammation.

These differences between studies and the different anti-inflammatory effects obtained may be due largely to a lack of knowledge of the effective dosage administration of bromelain indicated for postoperative inflammation in the extraction of third molars. Although further studies are required in this regard and various tests support the dose-dependent decrease in inflammation with bromelain (26), the analysis of these results seem to indicate a better response to third molar postoperative inflammation with lower doses but less spaced in time (25), than higher doses (150mg) at longer intervals (24 hours), as is the case in this study.

In summary and based on the results obtained, we can conclude that:

- The oral administration of bromelain may have beneficial effects in the control of postoperative inflammation and pain of impacted third molars, using an appropriate dose regimen, although this study has not highlighted these potential benefits.

- Further studies are needed to investigate the hypothesis that different dosing regimens of bromelain may be associated with a greater anti-inflammatory effect in the postoperative management of impacted third molars.
